# Factors influencing hospital high length of stay outliers

**DOI:** 10.1186/1472-6963-12-265

**Published:** 2012-08-20

**Authors:** Alberto Freitas, Tiago Silva-Costa, Fernando Lopes, Isabel Garcia-Lema, Armando Teixeira-Pinto, Pavel Brazdil, Altamiro Costa-Pereira

**Affiliations:** 1 CINTESIS - Center for Research in Health Technologies and Information Systems, CINTESIS, Portugal; 2 Department of Health Information and Decision Sciences, Faculty of Medicine, University of Porto, Porto, Portugal; 3 LIAAD - INESC Porto LA, Faculty of Economics, University of Porto, Porto, Portugal; 4 Faculty of Medicine, University of Porto, Al. Prof. Hern“ni Monteiro, 4200-319, Porto, Portugal

**Keywords:** Length of stay outliers, Administrative data, Hospital management, Case mix, Diagnosis related groups.

## Abstract

**Background:**

The study of length of stay (LOS) outliers is important for the management and financing of hospitals. Our aim was to study variables associated with high LOS outliers and their evolution over time.

**Methods:**

We used hospital administrative data from inpatient episodes in public acute care hospitals in the Portuguese National Health Service (NHS), with discharges between years 2000 and 2009, together with some hospital characteristics. The dependent variable, LOS outliers, was calculated for each diagnosis related group (DRG) using a trim point defined for each year by the geometric mean plus two standard deviations. Hospitals were classified on the basis of administrative, economic and teaching characteristics. We also studied the influence of comorbidities and readmissions. Logistic regression models, including a multivariable logistic regression, were used in the analysis. All the logistic regressions were fitted using generalized estimating equations (GEE).

**Results:**

In near nine million inpatient episodes analysed we found a proportion of 3.9% high LOS outliers, accounting for 19.2% of total inpatient days. The number of hospital patient discharges increased between years 2000 and 2005 and slightly decreased after that. The proportion of outliers ranged between the lowest value of 3.6% (in years 2001 and 2002) and the highest value of 4.3% in 2009. Teaching hospitals with over 1,000 beds have significantly more outliers than other hospitals, even after adjustment to readmissions and several patient characteristics.

**Conclusions:**

In the last years both average LOS and high LOS outliers are increasing in Portuguese NHS hospitals. As high LOS outliers represent an important proportion in the total inpatient days, this should be seen as an important alert for the management of hospitals and for national health policies. As expected, age, type of admission, and hospital type were significantly associated with high LOS outliers. The proportion of high outliers does not seem to be related to their financial coverage; they should be studied in order to highlight areas for further investigation. The increasing complexity of both hospitals and patients may be the single most important determinant of high LOS outliers and must therefore be taken into account by health managers when considering hospital costs.

## Background

### Length of stay outliers

A data object is called an outlier when it does not comply with the general behaviour of data [1]. Normally, outliers are very different or inconsistent with the remaining data. An outlier can be an error but can also result from the natural variability of data, and can hold important hidden information.

There is no universal technique for the detection of outliers; various factors have to be considered. Both in statistics and in machine learning it is possible to find many different methodologies [2-6]. Typically computer-based outlier analysis methods follow a statistical, a distance-based or a deviation-based approach [1].

Length of stay (LOS) is an important measure of resource utilization and, naturally, there are several systems for modelling and predicting LOS [7-10].

Specifically, the study of LOS outliers is essential for the management and financing of hospitals. The reimbursement of outliers is important either to protect patients that can a priori be more expensive and to protect hospitals from losses with uncommon cases [4]. LOS can in part explain hospital costs as there is a strong, not perfect, correlation between LOS and hospital costs [11,12]. A study in two public Spanish hospitals revealed that 4.8% of total patient discharges represent 15.4% of total LOS and 17.9% of total hospital costs [13]. In Portugal, costs are not available at the patient level.

Cots et al. [11] compared four different trimming methods for LOS outlier detection when cost is unknown. Their results showed that the use of the geometric mean plus two standard deviations had the highest level of agreement between LOS and cost and, simultaneously, exposed the major proportion of extreme outliers. The clear definition of LOS outliers is important because it underpins the proportion of cases identified as “outliers”. Because LOS distributions are skewed to the right, the log-transformation can help mitigate this skeweness [14].

In a different study, Cots et al. [15] analysed the relationship between hospital structural level and the presence of LOS outliers. In their study, outliers accounted for 4.5% of total hospital discharges. They verified that large urban hospitals have significantly more LOS outliers (5.6%) than medium size hospitals (4.6%) and small hospitals (3.6%). This result was previously shown by Bentes et al. [16].

Pirson et al. [17] studied cost outliers in a Belgian hospital and their results showed 6.3% of high resource use outliers and 1.1% for low resource use outliers. Instead of using geometric mean based trimming methods, they defined the trim points by the 75th percentile + 1.5*inter-quartile range for the selection of high cost outliers and the 25th percentile – 1.5*inter-quartile range for low cost outliers.

### Administrative data

Administrative databases may contain inaccurate data, but they are readily available, are relatively inexpensive and are widely used to assess resource use in hospital systems [18,19]. In some situations they can be the only source of information to look at a clinical question. Despite some existing problems [20-24], administrative data can, for instance, be used in the production of quality indicators, or for providing benchmarks of hospital activity [25-28]. Administrative data can provide the resources necessary to model an important percentage of the variation observed in hospital resource utilization [29].

### DRG in Portugal

DRG (Diagnosis Related Groups) is the most commonly used case mix system for hospital reimbursement and performance measurement. In Portugal it is used since 1990 [16] and has had positive impact on the productivity and technical efficiency of some diagnostic technologies [30].

The Portuguese government is both the main payer and provider of hospital care. Originally, key components of the DRG based inpatient resource allocation model were the DRG weights, hospital case mix indexes, hospital blended base rates and total number of discharge equivalents [16]. The model includes adjustments to account for outliers (low and high) and transfer cases.

Nowadays, annual hospital budgets are based on the expected number of inpatients and the types of admissions. A unique price is predetermined for all inpatient discharge (discharge equivalent) classified as medical DRGs. Similarly there are unique prices for other groups, such as surgical DRGs with programmed admission, surgical DRGs with emergency admission (admission that began at the emergency department), ambulatory surgery, outpatient visits, and emergency visits. Specific rates are applied for intensive care days (chronically ventilated patients). Special procedures and special ancillaries also had special rates in the past but are now priced by DRG. For some groups a different and specific case mix index is applied, specifically for medical DRGs, surgical DRGs with programmed admission, surgical DRGs with emergency admission, ambulatory surgery, and ambulatory medical procedures. Additional (or minor) hospital production is also contemplated in the annual contract (e.g., day case, days in units for physical medicine and rehabilitation, specific programs from the Ministry of Health). Some hospitals also receive an extra budget allocation to compensate for public service healthcare.

Since the start of this funding scheme in 2006, ^a^ the computing of case mix indexes ceased to account for high length of stay outliers. These cases are computed as normal cases thus doubly influencing the funding of hospitals as discharge equivalents are fewer and case mix indexes are lower in this calculus. For inpatients with third payer coverage, inpatient days above the high trim point are still covered by a fixed rate (per diem), regardless of the DRG in which the patient is grouped.

Bentes et al. [16] described the experience with the use of DRGs to fund Portuguese hospitals and verified that between 1989 and 1990 the number of long-stay patients (high LOS outliers) increased (+2.59%). Nevertheless, they stated that the apparent increase could not be real because, for many DRGs, the threshold was set at low levels. Despite that, central hospitals decreased the proportion of outliers (−4.8%). They also confirmed that larger hospitals had higher costs, even when accounting for their case mix.

### Teaching hospitals

Generally, the cost of care is higher in teaching hospitals than in non-teaching hospitals [31-36]. This can in part be explained by a more complex case mix, higher costs of labour, the cost of medical graduate education, or the use of more sophisticated technology [37].

The aim of this study was to find factors that explain length of stay outliers using available administrative data. These factors included the hospital group and the year of discharge.

## Methods

The administrative database associated with the Portuguese resource allocation system was the main source of data for this analysis. This database, with patient discharges between years 2000 and 2009, ^b^ included data from all the public acute care hospitals of the National Health Service (NHS). Data from private hospitals was not included in this study (representing around 15% of all inpatient stays in Portugal). The access to the data was provided by ACSS (Administração Central do Sistema de Saúde), the Ministry of Health’s Central Authority for Health Services.

### Pre-preparation

After some simple data validation, 0.2% of the cases were excluded and remained 9,253,087 inpatient stays for analysis. In this process of pre-preparation some simple validation rules were applied, with the rectification of data in some cases (when possible), and with its exclusion in other cases. Most of the excluded cases did not hold referential integrity, namely because of the use of incorrect ICD-9-CM (International Classification of Diseases, 9th Edition, Clinical Modification) diagnosis codes. For this analysis we only considered inpatient data; i.e., we excluded outpatient data (for instance ambulatory surgery).

### Data preparation

Length of stay (LOS) – is LOS a (high) outlier? No or Yes. Within each DRG (AP-DRG ^c^ version 21.0), and for every year, each length of stay (LOS) was classified as outlier or not using the (geometric mean + 2SD) as the trim point. We used the geometric mean plus two standard deviations as it could lead to a high level of agreement between costs and LOS, identifying the majority of extreme costs [11]. This method was used because the LOS distribution was approximately log-normal. This method is not useful for the detection of low outliers but that was not intended in this study.

Year of discharge – variable with 10 categories comprehending episodes with patient discharges between years 2000 and 2009.

Comorbidities – we applied the Elixhauser method, originally defined by Elixhauser et al. [38] and updated by Quan et al. [39]. Secondary diagnoses were used to determine the absence or presence of each one of the 31 comorbidities. The final score was the number of existing comorbidities.

Age – the age was recoded into 5 common groups, namely “[0 to 17] years”, “[18 to 45] years”, “[46 to 65] years”, “[66 to 80] years”, “More than 80 years”.

A-DRG complexity – using the relative weight of DRGs, we considered three groups [15], *low* (lower than the 1st quartile), *medium* (from 1st to 3rd quartile) and *high* (higher than 3rd quartile). We used adjacent DRGs (A-DRGs), which are rolled-up DRGs, because we wanted to exclude the information about comorbidities/complications and age breaks from the original DRG variable. The initial set of 668 DRGs (AP-DRG 21.0) was collapsed into 475 A-DRGs. For each A-DRG we considered the lower associated DRG cost-weight and used, for that, the prices published in the Portuguese *Diário da República*[40].

Readmission – patient readmission to the same hospital within 30 days, with categories “No readmission” or “Readmission”. We could not trace readmissions to other hospitals because the available patient identifier was unique to each hospital.

Admission and DRG type – 4 categories, the result from the combination of admission type (planned or emergency) with DRG type groups (surgical or non-surgical). “Planned and surgical”; “Planned and non-surgical”; “Emergency and surgical”; “Emergency and non-surgical”.

Discharge status – a dichotomous variable was used for patient discharge status: “Expired” (died in hospital) or “Discharged alive”.

Distance from residence to hospital – the distance was calculated in straight line and was further divided in 4 groups, “[0 to 4] km”, “]4 to 20] km”, “]20 to 60] km”, “More than 60 km”.

Hospital type – we used information available in several national publications (mostly from ACSS, ^d^ formerly IGIF) to define the three following hospital related variables:

“i)“ Administrative groups ^e^ – groups traditionally used in reports published by ACSS that categorizes hospitals in “Central hospital”, “District hospital” or “Level 1 district hospital”.

“ii)“ Economic groups ^f^ – another approach for grouping similar hospitals, in four groups. The original variable was defined according to hospital technology, technical differentiation and other factors. These factors included scale/specialities, complexity/case-mix and basic vs. intermediate structure. We created a new variable with two categories, “Group I” and “Groups II, III and IV”. Group I was different from the other category as it included specialized and complex hospitals, and hospitals with more technology.

“iii)” Teaching groups – we defined a trichotomic variable to distinguish between hospitals with undergraduate teaching, namely, large teaching hospitals (over 1,000 beds, corresponding to the 3 traditional and oldest teaching hospitals in Portugal), medium and small teaching hospitals (under 1,000 beds), and non-teaching hospitals.

### Statistical analysis

We used logistic regression models to examine the association of each variable with high LOS outliers. Afterwards we run a multivariable logistic regression with all the variables to compute adjusted odds ratios and their respective 95% confidence intervals. All the logistic regressions were fitted using generalized estimating equations (GEE) to take into account the dependence of observations due to the clustering effect by hospital.

The statistical analysis was performed using IBM SPSS Statistics version 20.0 and SAS version 9.1.

## Results

In the 9,253,087 cases studied we found 3.9% high LOS outliers. The median/mean LOS for these outliers was 25/35.5 days and 4/6.0 days for non-outliers. Being only 3.9% of the cases, outliers accounted for 19.2% of total discharge days.

Table 1 presents information about the variables studied and their influence in LOS outliers.

**Table 1:** Variables related with length of stay outliers^*^(N: 9,253,087)

***Variable Values***	***Cases (%)***	***Outliers (%)***	***Unadjusted OR***	***Adjusted OR (AOR)***	***95% CI for AOR lower - upper***
**LOS outlier**					
Not outlier	96.1				
Outlier	3.9				
**Admission and DRG type**					
Planned and non-surgical	8.9	3.1	1	1	
Planned and surgical	22.1	2.7	0.86	0.99	0.86 - 1.15
Emergency and non-surgical	55.5	3.9	1.26	1.46	1.32 - 1.62
Emergency and surgical	13.5	6.0	1.96	2.49	2.26 - 2.74
**Age**					
0 to 17 years	19.5	2.4	1	1	
18 to 45 years	27.7	2.7	1.13	0.99	0.88 - 1.12
46 to 65 years	20.7	4.2	1.76	1.53	1.33 - 1.76
66 to 80 years	22.4	5.5	2.32	1.78	1.52 - 2.08
More than 80 years	9.7	5.4	2.28	1.57	1.34 - 1.83
**Year of discharge**					
2000	10.3	3.9	1	1	
2001	10.4	3.6	0.92	0.90	0.87 - 0.94
2002	10.5	3.6	0.92	0.90	0.85 - 0.95
2003	10.7	3.9	1.00	0.97	0.90 - 1.04
2004	10.6	4.0	1.04	0.99	0.92 - 1.07
2005	10.8	3.8	0.98	0.93	0.86 - 1.00
2006	10.5	3.9	1.01	0.96	0.87 - 1.05
2007	10.4	3.9	1.02	0.95	0.85 - 1.05
2008	10.4	3.9	1.02	0.94	0.83 - 1.05
2009	5.4	4.3	1.12	1.01	0.90 - 1.14
**Number of comorbidities**					
(Elixhauser method)	**		1.85	1.41	1.34 - 1.49
**Discharge status**					
Discharged alive	95.6	3.7	1	1	
Expired	4.4	6.8	1.88	1.25	1.16 - 1.35
**A-DRG Complexity**					
Lower than 0.39	25.7	2.5	1	1	
From 0.39 to 1.02	49.3	4.1	1.66	1.14	1.07 - 1.21
Higher than 1.02	25.1	4.7	1.88	0.97	0.89 - 1.06
**Readmission**					
No readmission	93.5	3.8	1	1	
Readmission	6.5	4.9	1.32	1.21	1.13 - 1.29
**Hospital, administrative group**					
Level 1 district hospital	5.1	3.6	1	1	
District hospital	54.0	3.6	1.00	1.03	0.84 - 1.27
Central hospital	40.8	4.2	1.17	1.11	0.89 - 1.37
**Hospital, teaching groups**					
Non-teaching	70.7	3.7	1	1	
Medium-small teaching	16.0	4.1	1.13	1.03	0.92 - 1.16
Large teaching	13.4	4.5	1.25	1.17	1.03 - 1.33
**Hospital, economic group**					
Group II, III and IV	91.7	3.8	1	1	
Group I	8.3	4.4	1.17	1.13	0.94 - 1.37

* Proportion of cases, proportion of outliers, unadjusted odds ratios and adjusted odds ratios with 95% confidence intervals.

** 64% without any comorbidity.

### Descriptive data

The average LOS decreased from 7.30 days in year 2000 to 6.97 in year 2003 and, afterwards, continuously increased up to 7.26 in year 2009. The proportion of outliers decreased from 3.87% in year 2000 to 3.58% in year 2002 and, after that, continuously increased up to 4.32% in year 2009.

With 4.2%, central hospitals had more outliers than the other two groups (both with 3.6%). Hospitals from “Group I”, with 8.3% of the cases, had a higher proportion of outliers (4.4%) than other hospitals (3.8%). Considering teaching groups, large teaching hospitals had more outliers (4.5%) than other teaching hospitals (4.1%) and than non-teaching hospitals (3.7%).

For a better understanding of the hospitals included in this study we present in table 2 the case mix for each economic group, by year.

**Table 2:** Case mix* by hospital economic group and year

***Year of discharge***	Nbr. of hospitals^**^	***Group I (14 hosp.)***	***Group II (15 hosp.)***	***Group III (19 hosp.)***	***Group IV (47 hosp.)***
2000	94	1.434	1.141	0.813	0.790
2001	94	1.428	1.176	0.831	0.822
2002	91	1.444	1.206	0.834	0.834
2003	92	1.427	1.216	0.836	0.851
2004	87	1.447	1.249	0.865	0.874
2005	86	1.486	1.259	0.883	0.877
2006	81	1.441	1.327	0.905	0.889
2007	81	1.487	1.366	0.945	0.908

* Using relative weights from Portaria N. 132/2009.

** Hospitals with more than 100 hospitalizations in each year.

Considering other variables, we noted that outliers increased with age, from near 2.5% between 0 and 45 years, to about 5.5% for patients with more than 66 years. Within surgical DRG, we found 6% outliers for emergency admissions and 2.7% for planned admissions. Patient discharge status, readmissions and the number of comorbidities were also related with the increase in the proportion of outliers. We studied the evolution of discharge status over time (Table 3) and verified that both the proportion of patients “transferred to another hospital” and “discharged to home under care of organized home health service” is decreasing.

**Table 3:** Patient Discharge Status evolution by year

***Year of Discharge***	***N***	***Discharged to home or self-care(%)***	***Transferred to a Short-term General Hospital for Inpatient Care(%)***	***Discharged to home under care of organized home health service(%)***	***Left against medical advice(%)***	***Expired(%)***
2000	949,427	89.6	4.5	1.2	0.8	4.0
2001	959,895	89.8	4.3	1.0	0.8	4.1
2002	973,323	90.0	4.0	0.9	0.8	4.3
2003	991,799	90.3	3.6	1.1	0.7	4.3
2004	982,605	90.6	3.4	1.1	0.7	4.3
2005	995,770	90.5	3.5	0.8	0.7	4.6
2006	973,884	91.7	2.8	0.4	0.7	4.5
2007	965,576	91.3	3.0	0.4	0.7	4.7
2008	965,463	91.0	3.0	0.5	0.8	4.9
2009	495,345	90.9	2.8	0.7	0.7	4.9
**Total**	9,253,087	90.5	3.5	0.8	0.7	4.4

Apparently, the distance from residence to hospital had a slight influence in the incidence of outliers. With the increase of the distance from residence to hospital (comparing lower than 20 km with higher than 20 km) the proportion of outliers increased from 3.8% to 4.0%.

We also analysed the evolution of the readmission rate over the years and verified that it continuously increased between 2000 and 2007, from 5.0 to 7.2%, and decreased after that, with 6.4% in 2008 and 6.0% in 2009.

In tables 4 and Table 5 we can see the DRGs with higher and lower percentages of high LOS outliers. At the top we have DRG 236 (“FRACTURES OF HIP & PELVIS” with 8.4% of high LOS outliers and at the bottom we have DRG 429 (“ORGANIC DISTURBANCES & MENTAL RETARDATION”) with only 0.4% high LOS outliers. For this analysis we only considered DRGs with more than 10.000 discharges over the ten-year period.

**Table 4:** DRGs with higher percentages of high LOS outliers

DRG^*^	***DRG description***	***DRG weight***	***Number of stays***	***% outliers***
236	FRACTURES OF HIP & PELVIS	0.8932	13,994	8.4
203	MALIGNANCY OF HEPATOBILIARY SYSTEM OR PANCREAS	1.5427	29,369	7.2
584	SEPTICEMIA W MAJOR CC	2.1069	14,286	6.4
202	CIRRHOSIS & ALCOHOLIC HEPATITIS	1.2736	40,548	6.2
395	RED BLOOD CELL DISORDERS AGE >17	0.7048	33,459	6.2
112	PERCUTANEOUS CARDIOVASCULAR PROC W/O AMI,HEART FAILURE OR SHOCK	1.5594	16,895	6.2
205	DISORDERS OF LIVER EXCEPT MALIG,CIRR,ALC HEPA W CC	1.4658	13,259	6.1
206	DISORDERS OF LIVER EXCEPT MALIG,CIRR,ALC HEPA W/O CC	0.9732	22,295	6.1
133	ATHEROSCLEROSIS W/O CC	0.5287	12,244	6.1
179	INFLAMMATORY BOWEL DISEASE	0.9492	11,867	6.0
172	DIGESTIVE MALIGNANCY W CC	1.8601	36,407	6.0
078	PULMONARY EMBOLISM	0.9890	12,024	6.0
036	RETINAL PROCEDURES	1.3777	18,196	6.0

* DRGs both with more than 10.000 stays and 6% outliers (AP-DRG v.21).

**Table 5:** DRGs with lower percentages of high LOS outliers

DRG^*^	***DRG description***	***DRG weight***	***Number of stays***	***% outliers***
410	CHEMOTHERAPY	0.8616	102,193	1.9
163	HERNIA PROCEDURES AGE <18	0.6301	14,641	1.9
371	CESAREAN SECTION W/O CC	0.5825	218,924	1.8
373	VAGINAL DELIVERY W/O COMPLICATING DIAGNOSES	0.3948	454,185	1.6
119	VEIN LIGATION & STRIPPING	0.7212	83,038	1.3
629	NEONATE, BWT >2499 G, W/O SIGNIF OR PROC, W NORMAL NEWBORN DIAG	0.1118	775,53	1.0
430	PSYCHOSES	1.4619	65,188	0.6
429	ORGANIC DISTURBANCES & MENTAL RETARDATION	1.1290	15,537	0.4

* DRGs with more than 10.000 stays and less than 2% outliers (AP-DRG v.21.0).

### Logistic regression models

We used logistic regression for each variable, run a multivariable logistic regression will all the variables, and fitted all logistic regressions using generalized estimating equations (GEE). Table 1 shows the unadjusted odds ratios (ORs), and the adjusted ORs for the final multivariable logistic regression model.

Adjusted odds ratio for ‘Year of discharge’ decreased from 1 in 2000 to 0.9 in 2001 and 2002, that is, the proportion of outliers significantly decreased in this period (from 3.9% to 3.6%). After that, the odds ratio increased and, for 2009, it is nearly the same of 2000 (OR of 1.01 and 4.3% of outliers).

Considering hospital administrative groups we did not find significant differences between central hospitals when compared with the other two categories (adjusted OR = 1.11, CI_95%_: [0.89, 1.37]). For economic groups, “Group I” had an adjusted OR of 1.13 (also not statistically significant, CI_95%_: [0.94, 1.37]). For teaching groups, and after adjustment to other variables, large teaching hospitals had significantly more outliers than non-teaching hospitals (OR = 1.17, CI_95%_: [1.03, 1.33]).

The category “Emergency and surgical” in variable ‘Admission and DRG type’ was clearly more propitious for having outliers (OR = 2.49, CI_95%_: [2.26, 2.74]), when compared with the reference category “Planned and surgical”. Age categories “0 to 17 years” and “18 to 45 years” were quite similar and very different from the other 3 categories (with ORs between 1.5 and 1.8).

Comorbidities clearly influenced outliers (OR = 1.4, CI_95%_: [1.34, 1.49]). For “Expired” OR was 1.25 (CI_95%_: [1.16, 1.35]), that is, high LOS outliers were more likely to happen in episodes that resulted in death. For readmissions OR was 1.21 (CI_95%_: [1.13, 1.29]).

We found that the existing differences between the categories of the variable ‘Distance from residence to hospital’ had no importance and thus this variable was not included in the logistic regression analysis.

### Other trimming methods

We also studied LOS outliers using different trimming methods. With trim points defined by the 3rd quartile plus 1.5 times the inter-quartile range, we found 6.2% high outliers. Additionally we tried to identify low LOS outliers as in [17], using trim points defined by the 1st quartile minus 1.5 times the inter-quartile range. We found 5 DRGs with low LOS outliers (258 cases in 9,252,854 episodes and 677 distinct DRGs) with an overall percentage of 0.003. As for high outliers, we also used the traditional method, with trim points defined by the exponential function applied to the result of the arithmetic mean minus two standard deviations calculated over the log-normal distribution, and found a considerably higher proportion of low LOS outliers (2.028 versus 0.003%). Unlike the former, this traditional method always produces positive trim points as it is the result of the exponential function. In our case, we found 369 DRGs with low LOS outliers.

## Discussion

The study of LOS outliers is important as they are closely related to hospital costs. In fact, a small percentage of cases (3.9%) represent an important proportion in total inpatient days (19.2%).

This study shows that LOS outliers decreased in the beginning of last decade but significantly increased after that. We also verified that readmission rates increased between 2000 and 2007 and started to decrease after that. These factors, the increase in LOS outliers and high readmission rates, can contribute to an important portion of hospital costs and therefore should be considered by hospital managers and health policy makers.

We confirmed that emergent surgical admissions have significantly more outliers than planned surgical admissions. Moreover, patient age, the presence of specific comorbidities, and the discharge status, visibly influence LOS outliers.

Other important results are those related to hospital type. Using different hospital grouping variables, and after adjustments for the patients’ characteristics in the multivariate model, we only found statistically significant differences between teaching groups. All the three hospital related variables seem to have influence in LOS outliers but only large undergraduate teaching hospitals (in hospital teaching groups) have significantly more outliers (4.5%). In the other groups, we found that central hospitals (administrative groups) have more outliers than others hospitals, and that hospitals with higher technology, specialized and complex (Group I, economic groups) have also more outliers.

The proportion of LOS outliers in this study is lower than that found in a study in Catalonia (Spain) for discharges in 1998 (4.5% outliers) [15]. In another study [17] the proportion of high outliers is even higher but, in that case, they used hospital real costs and not an approximation to cost through LOS. These differences cannot be easily explained given the possible differences in hospital case mixes of these studies, among other structural or health policy differences.

The proportion of outliers does not seem to be related to their financial coverage: if anything, the proportion of high length of stay outliers increased in the years they were disregarded in public funding, from 3.9% in 2006 to 4.3% in 2009. The evolution of case mix indexes in the several economic groups may support this result: in all groups, case mix indexes are increasing over the years although in Group 1 this evolution is quite irregular and the index even diminishes in 2006, picking up in the following years. If hospitals had been able to easily control the volume of high LOS outliers, then it would be expectable to find some influence in the evolution of the case mix over the years. In fact, after 2006, case mix indexes consistently increased despite the lack of specific funding for inpatient days above the high trim point.

Actually, hospital doctors normally try to avoid the long duration of the stays. They are typically not conscious of any resulting extra value on funding, but they are aware of the greater likelihood of inpatient complications, being the decrease of the average LOS one of their main concerns.

A greater hospital complexity is generically related to an increase in the proportion of outliers. Nevertheless, we may find hospitals in the same hospital group (with similar complexity) with considerable differences in the proportion of outliers for specific DRGs. As an example, for DRG 236 (FRACTURES OF HIP & PELVIS) the global proportion of outliers is 8.4%, but there is a wide hospital intra-group variation, ranging from one hospital with 4.2% to other with 18.0% high outliers. Under these circumstances, we could argue that at least a part of these outlier cases could be prevented, and so, extra funding would be a reward of poor patient management.

Using the daily price published in *Diário da República*[40] (83.3€), we estimated that hospitals received between 2.2% and 1.7% of their budget due to high LOS outliers in the period 2000 to 2005 and, after that, they potentially were not reimbursed for 1.59% (2006), 1.63% (2007), 1.54% (2008), and 1.64% (2009) of their costs.

Better clinical coding with fewer errors over the time could be one of the reasons for the evolution of the proportion of outliers. To examine this possibility we picked up and analysed several cases with extreme outliers in one central hospital. Associated patient records were audited by a medical doctor and no errors were found. Better clinical coding can influence the quality of data but it is not the main reason for the variance of outliers over time.

Administrative databases were created mainly to serve a billing role and some limitations arise directly from this purpose. They are a valuable research tool but their limitations should be kept in mind. For instance, the number of available variables is limited; the quality of coding is not uniform over time or between different hospitals, among other data quality problems.

## Conclusions

Resources are scarce and need to be properly distributed and clearly justified. Outliers have influence in hospital costs and therefore should be considered in the financing of hospitals, although, case review should also be implemented to try to avoid preventable outlier cases. It is important to be aware of this kind of information for hospital planning and policy. The increasing complexity of both hospitals and patients may be the single most important determinant of high LOS outliers and must therefore be taken into account in the future by health managers when considering hospital costs.

## Endnotes

^a^Circular Informativa N. 3 of 24/08/2006 from ACSS (formerly IGIF)

^b^Year 2009 is not complete (includes only records for the first half of that year)

^c^All Patient Diagnosis Related Groups

^d^http://www.acss.min-saude.pt

^e^Portaria N. 281/2005 of 17/03/2005

^f^Relatório de Retorno Nacional – 2006; Unidade Operacional de Financiamento e Contratualização, ACSS (formerly IGIF)

## Competing interests

The authors have no competing interests to disclose.

## Authors’ contributions

AF conceived the study, performed statistical analyses, and drafted the manuscript. TSC assisted in data preparation and in drafting the manuscript. FL and IL assisted in ICD-9-CM and other classification/coding aspects, and in drafting the manuscript. ATP assisted in the statistical analyses. PB assisted in drafting the manuscript. ACP assisted in drafting the manuscript. All authors have approved the final version of the manuscript.

## Pre-publication history

The pre-publication history for this paper can be accessed here:

http://www.biomedcentral.com/1472-6963/12/265/prepub

## References

[B1] Han J Kamber M Data Mining: Concepts and Techniques 2000 Morgan Kaufmann, 1st edition

[B2] Rosner B Percentage Points for a Generalized ESD Many-Outlier Procedure Technometrics 1983 25 2 165 172 10.1080/00401706.1983.10487848

[B3] Hodge VJ Austin J A survey of outlier detection methodologies Artificial Intelligence Review 2004 22 85 126

[B4] Lee AH Xiao J Vemuri SR Zhao Y A discordancy test approach to identify outliers of length of hospital stay Statistics in Medicine 1998 17 2199 2206 10.1002/(SICI)1097-0258(19981015)17:19<2199::AID-SIM917>3.0.CO;2-2 9802178

[B5] Podgorelec V, Heričko M, Rozman I: Improving mining of medical data by outliers prediction. In: Proceedings of the 18th IEEE Symposium on Computer-Based Medical Systems: 23–24 Dublin, Ireland IEEE Computer Society June 2005 2005 91 96

[B6] Ramaswamy S, Rastogi R, Shim K: Efficient algorithms for mining outliers from large data sets. In: Proceedings of the ACM SIGMOD International Conference on Management: 14–19 May 2000; Dallas, Texas, United States ACM Press 2000 2000 427 438

[B7] Abe T Toyabe S-i, Cao P, Kurashima S, Akazawa K: Development of a simulation program for estimating hospital incomes under the prospective payment system Computer Methods and Programs in Biomedicine 2005 80 271 276 10.1016/j.cmpb.2005.09.003 16219386

[B8] Kraft MR Desouza KC Androwich I Data mining in healthcare information systems: Case study of a veterans’ administration spinal cord injury population 2003 IEEE Computer Society, In: Proceedings of the 36th Hawaii International Conference on System Sciences: 6–9 January 2003; Hawaii

[B9] Marshall A Vasilakis C El-Darzi E Length of stay-based patient flow models: recent developments and future directions Health Care Management Science 2005 8 213 220 10.1007/s10729-005-2012-z 16134434

[B10] Shu-Kay N McLachlan GJ Lee AH An incremental EM-based learning approach for on-line prediction of hospital resource utilization Artificial Intelligence in Medicine 2006 36 3 257 267 10.1016/j.artmed.2005.07.003 16213695

[B11] Cots F Elvira D Castells X Relevance of outlier cases in case mix systems and evaluation of trimming methods Health Care Management Science 2003 6 27 35 10.1023/A:1021908220013 12638924

[B12] Polverejan E Gardiner JC Bradley CJ Holmes-Rovner M Rovner D Estimating mean hospital cost as a function of length of stay and patient characteristics Health Economics 2003 12 11 935 947 10.1002/hec.774 14601156

[B13] Cots F Elvira D Castells X Dalmau E Medicare’s DRG-weights in a European environment: the Spanish experience Health Policy 2000 52 31 47 11010224 10.1016/s0168-8510(99)00074-3

[B14] Diehr P Yanez D Ash A Hornbrook M Lin DY Methods for analyzing health care utilization and costs Annual Reviews of Public Health 1999 20 125 144 10.1146/annurev.publhealth.20.1.125 10352853

[B15] Cots F Mercadé L Castells X Salvador X Relationship between hospital structural level and length of stay outliers: Implications for hospital payment systems Health Policy 2004 68 159 168 10.1016/j.healthpol.2003.09.004 15063016

[B16] Bentes ME Urbano JA Carvalho MC Tranquada MS Using DRGs to fund hospitals in Portugal: An evaluation of the experience In: Diagnosis Related Groups in Europe: Uses and perspectives 993 Edited by Casas M, Wiley MM, Berlin: Springer-Verlag 173 182

[B17] Pirson M Dramaix M Leclercq P Jackson T Analysis of cost outliers within APR-DRGs in a Belgian general hospital: Two complementary approaches Health Policy 2006 76 13 25 10.1016/j.healthpol.2005.04.008 15921818

[B18] Iezzoni LI Assessing quality using administrative data Annals of Internal Medicine 1997 127 666 674 9382378 10.7326/0003-4819-127-8_part_2-199710151-00048

[B19] Torchiana D Meyer G Use of administrative data for clinical quality measurement Journal of Thoracic and Cardiovascular Surgery 2005 129 6 1222 1224 10.1016/j.jtcvs.2005.02.020 15942560

[B20] Calle J Saturno P Parra P Rodenas J Perez M Eustaquio F Aguinaga E Quality of the information contained in the minimum basic data set: results from an evaluation in eight hospitals European Journal of Epidemiology 2000 16 11 1073 1080 10.1023/A:1010931111115 11421479

[B21] Powell AE Davies HTO Thomson RG Using routine comparative data to assess the quality of health care: understanding and avoiding common pitfalls Quality and Safety in Health Care 2003 12 122 128 10.1136/qhc.12.2.122 12679509 PMC1743685

[B22] Sutherland JM Botz CK The effect of misclassification errors on case mix measurement Health Policy 2006 79 195 202 10.1016/j.healthpol.2005.12.012 16439035

[B23] Williams J Mann R Hospital episode statistics: time for clinicians to get involved? Clinical Medicine 2002 2 34 37 11871636 10.7861/clinmedicine.2-1-34 PMC4953168

[B24] Freitas A Silva-Costa T Marques B Costa-Pereira A Implications of Data Quality Problems within Hospital Administrative Databases 2010 In IFMBE Proceedings. Volume 29. Edited by Bamidis PD, Pallikarakis N, Springer Berlin Heidelberg 823 826

[B25] Jarman B Gault S Alves B Hider A Dolan S Cook A Hurwitz B Iezzoni LI Explaining differences in English hospital death rates using routinely collected data BMJ 1999 318 7197 1515 1520 10.1136/bmj.318.7197.1515 10356004 PMC27892

[B26] Scott I Youlden D Coory M Are diagnosis specific outcome indicators based on administrative data useful in assessing quality of hospital care? Quality and Safety in Health Care 2004 13 32 39 10.1136/qshc.2002.003996 14757797 PMC1758063

[B27] Zhan C Miller MR Administrative data based patient safety research: a critical review Quality and Safety in Health Care 2003 12 58 63 10.1136/qhc.12.1.58 14645897 PMC1765777

[B28] Freitas A Costa T Marques B Gaspar J Gomes J Lopes F Lema I A Framework for the Production and Analysis of Hospital Quality Indicators In Information Technology in Bio- and Medical Informatics, Lecture Notes in Computer Science 2011 Volume 6865. Edited by Böhm C, Khuri S, Lhotská L, Pisanti N, Springer Berlin Heidelberg 96 105

[B29] Evans E Imanaka Y Sekimoto M Ishizaki T Hayashida K Fukuda H Oh EH Risk adjusted resource utilization for AMI patients treated in Japanese hospitals Health Economics 2007 16 4 347 359 10.1002/hec.1177 17031780

[B30] Dismuke C Sena V Has DRG payment influenced the technical efficiency and productivity of diagnostic technologies in Portuguese public hospitals? An empirical analysis using parametric and non-parametric methods Health Care Management Science 1999 2 2 107 116 10.1023/A:1019027509833 10916607

[B31] Dalton K Norton E Kilpatrick K A longitudinal study of the effects of graduate medical education on hospital operating costs Health Services Research 2001 35 6 1267 1291 11221819 PMC1089190

[B32] López-Casasnovas G Sáez M The impact of teaching status on average costs in Spanish hospitals Health Economics 1999 8 641 651 10.1002/(SICI)1099-1050(199911)8:7<641::AID-HEC475>3.0.CO;2-L 10544329

[B33] Mechanic R Coleman K Dobson A Teaching hospital costs: implications for academic missions in a competitive market JAMA 1998 280 11 1015 1019 10.1001/jama.280.11.1015 9749488

[B34] Rosko M Performance of US teaching hospitals: a panel analysis of cost inefficiency Health Care Management Science 2004 7 1 7 16 14977089 10.1023/b:hcms.0000005393.24012.1c

[B35] Shanahan M Loyd M Roos N Brownell M A comparative study of the costliness of Manitoba hospitals Medical Care 1999 37 6 101 122 10.1097/00005650-199906001-00011 10409003

[B36] Yuan Z Cooper G Einstadter D Cebul R Rimm A The association between hospital type and mortality and length of stay: a study of 16.9 million hospitalized Medicare beneficiaries. Medical Care 2000 38 2 231 245 10.1097/00005650-200002000-00012 10659696

[B37] Taylor DJ Whellan D Sloan F Effects of admission to a teaching hospital on the cost and quality of care for Medicare beneficiaries The New England Journal of Medicine 1999 340 4 293 299 10.1056/NEJM199901283400408 9920955

[B38] Elixhauser A Steiner C Harris R Coffey R Comorbidity measures for use with administrative data Medical Care 1998 36 8 27 10.1097/00005650-199801000-00004 9431328

[B39] Quan H Sundararajan V Halfon P Fong A Burnand B Luthi JC Saunders LD Beck CA Feasby TE Ghali WA Coding algorithms for defining Comorbidities in ICD-9-CM and ICD-10 administrative data Medical Care 2005 43 11 1130 9 10.1097/01.mlr.0000182534.19832.83 16224307

[B40] da Saúde M Tabela Nacional de Grupos de Diagnóstico Homogéneo (anexo II) Portaria n 567/2006, Diário da República – I Série-B, de 12 de Junho de 2006 113 4173 4203

